# Morphofunctional Spaces from the Astragalus: Exploring Angular Excursions and Mechanical Efficiency in *Caraguatypotherium munozi* (Notoungulata, Mesotheriidae)

**DOI:** 10.3390/biology14091290

**Published:** 2025-09-18

**Authors:** Paul Medina-González

**Affiliations:** Departamento de Kinesiología, Facultad de Ciencias de la Salud, Universidad Católica del Maule, Talca 3480112, Chile; pmedina@ucm.cl or paulmedinagonzalez@gmail.com

**Keywords:** astragalus, functional morphology, stance-phase locomotion, Miocene mammals, comparative biomechanics

## Abstract

The astragalus is a key biomechanical link between the autopodium and limb in mammals, modulating stability and mobility during stance. Its morphology provides reliable proxies for inferring posture, body mass, and locomotor behavior in extinct taxa. Mesotheriids (Notoungulata, Mesotheriidae) have traditionally been regarded as fossorial generalists, yet their functional diversity remains poorly tested. The astragali of *Caraguatypotherium munozi* (Miocene, Chile) and *Trachytherus spegazzinianus* (early Miocene, Argentina) were analyzed, integrating osteological measurements and functional indices to explore their locomotor ecology. Principal component analyses were performed to compare their morphofunctional spaces with those of 38 extant terrestrial mammals grouped by posture, body mass, top speed, and locomotor habit. Total Angular Excursion (TAE) and Angular Efficiency Index (AEI) were estimated using a comparative dataset of 182 terrestrial mammals spanning 15 taxonomic orders, focused on stance-phase mechanics during comfortable locomotion. *C. munozi* shows a deeper trochlea and moderately expanded navicular facets, whereas *T. spegazzinianus* presents a shallower trochlea and narrower articular proportions. Despite these morphological differences, both species share broadly overlapping stance-phase kinematic ranges, reflecting a conserved plantigrade locomotor module optimized for stability and energy-efficient weight support. These findings challenge the view of mesotheriids as functionally uniform and highlight ecological diversification through subtle morphological adjustments within a constrained locomotor framework.

## Simple Summary

Understanding how extinct mammals moved provides essential clues about their ecological roles and adaptive strategies. In mammals, the astragalus (ankle bone) is crucial for locomotion because it transfers forces between the leg and the foot, balancing stability and mobility during walking. This study focuses on the examination of the astragali of two extinct South American mammals, *Caraguatypotherium munozi* and *Trachytherus spegazzinianus*, both members of Mesotheriidae—a group traditionally considered fossorial generalists. By combining bone measurements with functional indices, the performance of the ankle during the stance phase of walking was estimated. The results reveal marked ecological differences: *C. munozi* occupied a terrestrial–scansorial niche, showing moderate joint mobility and mechanical efficiency consistent with climbing tendencies, whereas *T. spegazzinianus* exhibited a semifossorial profile with a more stability-oriented ankle suited for digging. Despite these ecological differences, both species shared similar stance-phase kinematic ranges, reflecting a conserved plantigrade locomotor module optimized for stability and energy-efficient weight support. These findings challenge the traditional view of mesotheriids as functionally uniform and demonstrate that closely related species could achieve ecological diversification through subtle morphological adjustments within a shared locomotor framework.

## 1. Introduction

Understanding how anatomical form reflects biological function remains a cornerstone of functional morphology. Classical frameworks conceptualize traits as *form–function complexes* shaped by natural selection under specific ecological pressures [1]. From this perspective, skeletal structures are not static remnants but biomechanical solutions shaped by evolutionary trade-offs between structural integrity, mechanical performance, and ecological demands [2]. Such an approach is particularly crucial in paleontology, where the absence of extant analogs requires inferring function indirectly through mechanical analogies and ecomorphological proxies [3,4,5].

One of the most conspicuous examples of form–function integration in vertebrates is limb posture, which strongly influences locomotor mechanics and ecological performance. Mammals span a continuum from plantigrade to digitigrade and unguligrade postures, reflecting trade-offs between stability, energy economy, and cursoriality [6,7,8]. Plantigrady, generally considered primitive, maximizes substrate contact and mediolateral stability, favoring low-speed terrestriality or fossoriality, whereas digitigrady and unguligrady increase parasagittal limb alignment, reducing mechanical stresses and enabling efficient high-speed locomotion [9,10]. Interestingly, plantigrade postures persist even in some large mammals (e.g., ursids), as stability and force generation can outweigh the selective pressures for speed [7].

Among postcranial elements, the astragalus (talus) has emerged as a critical ecomorphological proxy for reconstructing locomotor behavior in extinct mammals. Acting as the primary hinge between the tibia and foot, its articular surfaces integrate weight-bearing, postural stability, and mobility [6,11]. Deep, symmetric trochleae restrict motion to parasagittal planes, characteristic of digitigrade and cursorial taxa, whereas shallower or asymmetric trochleae allow greater mediolateral excursion, typical of plantigrade species [6,11]. Astragalar dimensions also scale predictably with body mass, with the width of the tibial trochlea serving as a robust estimator across extant mammals [12]. However, because astragalar morphology reflects multiple interacting demands—support, propulsion, and substrate interaction—its functional signal often overlaps among postural categories [6]. For this reason, a multi-proxy functional framework that integrates structural morphology with biomechanical performance indices, such as joint angular excursions and mechanical efficiency, is essential to refine morphofunctional interpretations.

The stance phase of walking provides a particularly informative functional window, as limbs are loaded and directly constrained by ground reaction forces. Joint angular excursions during this phase encode how internal musculoskeletal architecture responds to external mechanical constraints, defining support, braking, and propulsive roles [9,10,13,14]. Moreover, stance-phase mechanics set the kinematic and energetic boundary conditions for the swing phase, influencing stride length, limb trajectories, and overall mechanical economy [15]. Thus, quantifying stance-phase angular excursions and deriving mechanical efficiency indices offers a robust, comparative framework for inferring locomotor strategies in extinct mammals.

Mesotheriids (*Notoungulata*, *Mesotheriidae*) provide an exceptional model for applying this integrative approach. These endemic South American ungulates, which diversified between the Oligocene and Late Pliocene, display remarkable morphological and ecological diversity, with body mass estimates ranging from ~22 to >400 kg [16], and inferred locomotor behaviors spanning terrestrial ambulatory to semifossorial strategies [17,18]. However, functional interpretations remain contentious due to the absence of extant ecological analogs [17]. The prevailing hypothesis portrays mesotheriids as generalist terrestrial walkers with predominantly scratch-digging capacities, yet this assumption has seldom been tested using biomechanical approaches.

Among mesotheriids, *Caraguatypotherium munozi* stands out as a particularly informative case study. This medium-sized Miocene species from Chile occupied an intermediate geographic range between northern and southern mesotheriid populations [19]. Phylogenetically, *Caraguatypotherium* is more closely related to *Plesiotypotherium*, a genus with less robust forelimbs and fewer fossorial specializations, whereas *Trachytherus spegazzinianus*—a basal trachytheriine—ref. [20] has been consistently interpreted as a semifossorial scratch-digger based on forelimb robusticity and generalized astragalar morphology [17,18].

*Caraguatypotherium munozi* derives from the lower Huaylas Formation in northernmost Chile, near the confluence of Quebradas Belén and Lupica in the Caragua area (Arica–Parinacota). Stratigraphic relationships, synsedimentary deformation, and intercalated volcanic units bracket deposition between ~11.4 and ~10.7 Ma [19,20]. By contrast, *Trachytherus spegazzinianus* is an early-diverging mesotheriid from Deseadan horizons (late Oligocene; ~29.4–25.8 Ma) in central Andean localities around the “Arica bend” (southern Peru–western Bolivia–northern Chile) and farther south in South America, with occurrences documented in Bolivia and Argentina [17,20]. These differences in age and depositional setting provide the context for interpreting astragalar morphology and hindlimb function.

This study aims to test the hypothesis that not all mesotheriids conformed to a generalized semifossorial pattern. Specifically, this study explores whether *C. munozi* occupied a distinct stance-phase morphofunctional space biased toward stability and moderate mobility, contrasting with the more generalized semifossorial strategy typical of early mesotheriids such as *T. spegazzinianus*. By reconstructing astragalar morphofunctional spaces through angular excursion and mechanical efficiency indices, this study provides new insights into locomotor diversity and ecological disparity within Mesotheriidae.

## 2. Materials and Methods

### 2.1. Specimens and Comparative Sample

This study analyzed astragalar indicators to infer stance-phase locomotor adaptations in *Caraguatypotherium munozi*. The dataset comprised 40 astragali, including 38 extant terrestrial mammals with well-documented locomotor patterns and two mesotheriid taxa. The extant sample included primates (*n* = 15), artiodactyls (*n* = 7), carnivores (*n* = 10), diprotodonts (*n* = 2), dermopterans (*n* = 1), didelphimorphians (*n* = 1), rodents (*n* = 1), and scandentians (*n* = 1), representing a wide range of body masses, postures, and locomotor habits.

**Figure 1:**
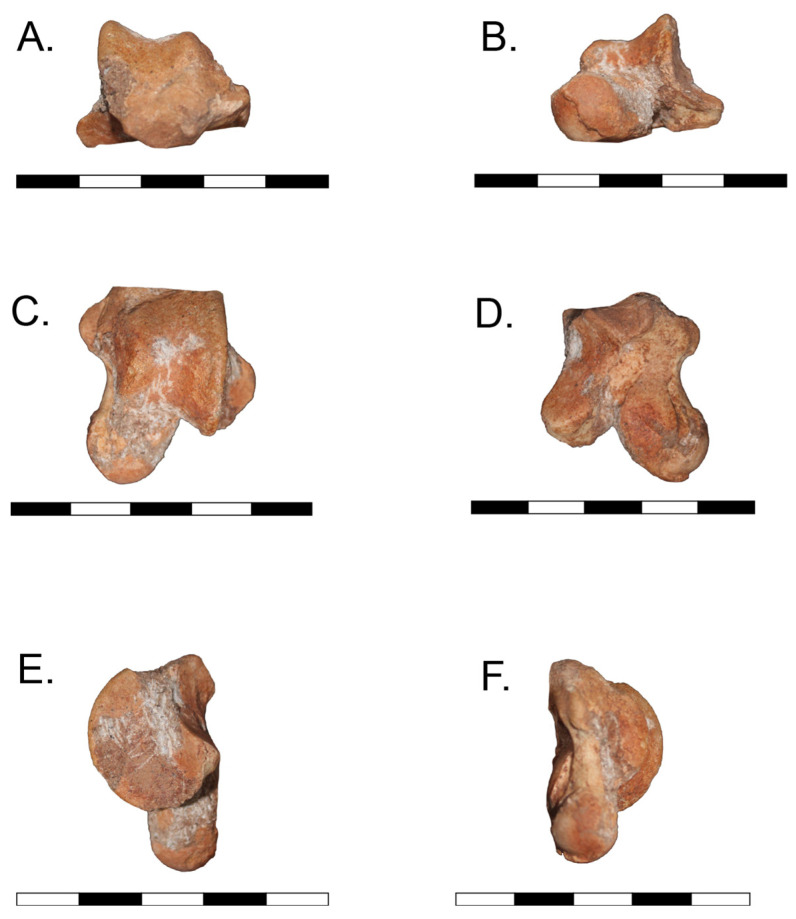
Astragalus of *Caraguatypotherium munozi* (PTY-050, Laboratorio de MacroPaleo, Universidad Austral de Chile), shown in six views: (**A**) anterior, (**B**) posterior, (**C**) dorsal, (**D**) plantar, (**E**) medial, and (**F**) lateral. Scale bar = 50 mm.

The material of *Caraguatypotherium munozi* consisted of a referred astragalus curated at the Laboratorio de Macropaleontología, Universidad Austral de Chile (UACh); internal label “PTY-050” (uncatalogued, Figure 1). For comparative purposes, scaled photographs and measurements of *Trachytherus spegazzinianus* (UF 172437) were obtained from the literature [17]. A complete list of specimens and associated metadata is provided in Supplementary File S1.

### 2.2. Osteological Indicators and Measurements

Linear measurements for the *Caraguatypotherium munozi* astragalus were obtained directly on the physical specimen using digital calipers (±0.01 mm). Each variable was measured three times by the same observer, and the mean value was reported when the measurement error was <5%. These values were further cross-validated against calibrated photographs of the same specimen. For all other specimens, and for all angular and area variables (including those of *C. munozi*), measurements were derived exclusively from calibrated photographs. Landmark definitions followed established protocols [6,11,21].

The photographic measurement protocol demonstrated excellent inter-rater reliability (ICC = 0.977; 95% CI = 0.975–0.988; *p* < 0.001; *n* = 38) and strong criterion-related validity against direct caliper measurements (R = 0.964; 95% CI = 0.921–0.988; *p* < 0.001; *n* = 27; R^2^ = 0.929) [22]. These metrics confirm that the implemented protocol achieves a high standard of measurement quality, ensuring consistent and valid estimates of linear, angular, and area variables across specimens.

Image-based measurements used photographs from three sources: (i) in-house images taken at the Laboratorio de Anatomía Comparada, Universidad Austral de Chile (UACh) with a 50 mm digital scale in view; (ii) the online osteology database BoneID; and (iii) peer-reviewed publications (PDFs/high-resolution figures). Pixel-to-millimeter calibration was performed in Digimizer (version 2023.1.1, MedCalc Software Ltd., Acacialaan 22, Ostend, Belgium, 2023) and Tracker (version 6.1.6, Open Source Physics, Davidson College, 405 N. Main Street, Davidson, NC, USA, 2024). For UACh photographs, calibration was based on the embedded 10 mm scale bar; for BoneID and published figures, calibration relied on the printed scale bar or, when absent, on a reported linear dimension in the figure, caption, or text. Only approximately orthogonal views were used. Orientation was standardized by aligning the astragali according to their anatomical position in quadrupedal stance, and images were consistently cropped to ensure comparability across specimens. Images lacking a reliable calibration reference were excluded.

The astragalus was selected as the primary osteological proxy because it functions as the biomechanical hinge of the ankle, reflecting posture, weight-bearing demands, and stance-phase mobility. Measurement protocols followed standardized definitions [6,11,12,23] for: astragalar maximum width within the trochlea (AMWT), astragalar navicular surface length (ANSL), astragalar tibial–tarsal angle (ATTA), astragalar tibial articular area (ATAA), astragalar calcaneal articular area (ACAA; subdivided into sustentacular and ectal facets), astragalar navicular articular area (ANAA), lateral trochlear length (LTL), and medial trochlear length (MTL).

Angles were measured on calibrated images by constructing baselines between anatomical landmarks and recording the arc between them (degrees, one decimal). Areas (ATAA, ACAA—sustentacular and ectal—and ANAA) were delineated as polygon ROIs (mm^2^). Each angle and area were digitized three times and averaged.

The astragalar tibial–tarsal angle (ATTA, also referred to as the head orientation angle, HOA) describes the mediolateral orientation of the astragalar head relative to the trochlea. Larger angles indicate increased transverse mobility, typically associated with plantigrade or scansorial strategies, whereas lower angles reflect parasagittal alignment and reduced mediolateral motion, characteristic of cursorial taxa [6,11].

**Figure 2:**
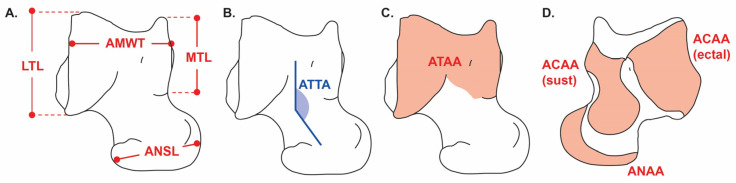
Osteological measurements of the astragalus of *Caraguatypotherium munozi* in dorsal (**A**–**C**) and plantar (**D**) views (modified from Ginot et al., 2016) [11]. (**A**) Linear measurements. (**B**) Angular measurement. (**C**,**D**) Articular surface areas. Measured variables include astragalar maximum width within the trochlea (AMWT), lateral trochlear length (LTL), medial trochlear length (MTL), astragalar navicular surface length (ANSL), astragalar tibial–tarsal angle (ATTA, also referred to as the head orientation angle, HOA), astragalar tibial articular area (ATAA), astragalar calcaneal articular area (ACAA; subdivided into sustentacular and ectal facets), and astragalar navicular articular area (ANAA).

All variables, landmarks, baselines, arcs, and area ROIs are illustrated in Figure 2 (dorsal and plantar views). Supplementary File S1 includes a “Source of origin” column indicating Direct (caliper), UACh photo, BoneID, or Literature for each record, together with the raw measurement files in CSV format for full traceability.

### 2.3. Functional Indices of the Astragalus

Locomotor function was assessed by deriving five functional indices from linear, and articular surface measurements of the astragalus. These indices are well established as proxies for postural stability, mediolateral mobility, and weight-bearing efficiency in extant and extinct mammals [6,11,12,23].

The Trochlear Depth Index (TDI) quantified relative trochlear depth as a proxy for parasagittal motion restriction and overall stability. It was calculated as the mean height of the medial and lateral trochlear ridges (LTL and MTL) divided by the astragalar maximum width of the trochlea (AMWT):

$$TDI=\frac{\left(LTL+MTL\right)/2}{AMWT}$$

Higher TDI values indicate deeper trochleae and stronger parasagittal stabilization, typical of terrestrial or semifossorial locomotion, whereas lower values suggest greater mediolateral mobility, characteristic of plantigrade or scansorial taxa [6,11].

The Navicular Facet Proportions (NFP) were calculated as the ratio between the astragalar navicular surface length (ANSL) and the navicular articular area (ANAA):

$$NFP=\frac{ANSL}{ANAA}$$

Larger NFP values are associated with enhanced multidirectional ankle mobility, whereas smaller values correspond to restricted parasagittal motion [11].

Relative articular surface areas were also estimated to explore the functional balance between stability and mobility while controlling for size effects. The Trochlear Relative Articular Area (TRAA) was calculated as the tibial articular area (ATAA) divided by the total articular surface area (ATAA + ANAA + ACAA):

$$TRAA=\frac{ATAA}{ATAA+ANAA+ACAA}$$

High TRAA values reflect increased parasagittal stability and weight-bearing efficiency. Conversely, the Navicular Relative Articular Area (NRAA), calculated as ANAA divided by the total articular area, indicates multidirectional mobility, with higher values typical of plantigrade or scansorial taxa:

$$NRAA=\frac{ANAA}{ATAA+ANAA+ACAA}$$

Finally, the Calcaneal Relative Articular Area (CRAA), calculated as the proportion of the calcaneal articular area (ACAA, including ectal and sustentacular facets) relative to the total articular area, is associated with force transmission and digging performance; higher CRAA values are expected in semifossorial taxa:

$$CRAA=\frac{ACAA}{ATAA+ANAA+ACAA}$$

These five indices were subsequently integrated into a multivariate framework (PCA) to reconstruct stance-phase morphofunctional spaces, enabling a direct functional comparison between *Caraguatypotherium munozi* and *Trachytherus spegazzinianus*.

### 2.4. Data Normalization and Preprocessing for Multivariate Analysis

To ensure comparability across specimens of different sizes, all variables were expressed in size-free form prior to multivariate analysis.

(i) Linear dimensions were normalized relative to the astragalar maximum trochlear width (AMWT), yielding ratios such as LTL_norm = LTL/AMWT, MTL_norm = MTL/AMWT, and ANSL_norm = ANSL/AMWT.
(ii) Articular surface areas were relativized to the total articular area (TAA = ATAA + ANAA + ACAA), producing ATAA_rel, ANAA_rel, and ACAA_rel.
(iii) The astragalar tibial–tarsal angle (ATTA, °) is inherently size-independent and was included without transformation.
(iv) Functional variables (e.g., Trochlear Depth Index, TDI, and other biomechanical ratios) are already normalized expressions of morphology, and were therefore included directly after z-standardization.

Prior to PCA, all size-free variables (normalized lengths, relative areas, ATTA, and functional variables) were z-standardized (X − μ/σ) using the mean (μ) and standard deviation (σ) of the extant dataset. Fossil mesotheriids were scaled using the same parameters to maintain comparability. PCA was performed on the Pearson correlation matrix. Full datasets corresponding to each stage (raw values, normalized ratios, functional indices, and z-standardized variables), together with the scaling parameters (μ, σ) from the extant sample, are provided in Supplementary File S1.

### 2.5. Stance-Phase Angular Excursion Analysis

To functionally position *Caraguatypotherium munozi* within the morphospace of extant mammals, its osteological indicators were compared with stance-phase angular excursion data compiled in a comprehensive Zenodo dataset [24]. This dataset comprises 182 terrestrial mammal species spanning 15 taxonomic orders, focusing on the stance phase during comfortable locomotion. It integrates original video analyses from publicly available footage (*n* = 81) and supplementary data extracted from the published literature (*n* = 101). All procedures involving video and frame-sequence analysis were approved by the Institutional Committee for Animal Care and Use at Universidad Católica del Maule, Chile (CICUAL-UCM, 2023).

Joint angles were measured at three key stance-phase events—touchdown (TD), midstance (MS), and toe-off (TO)—representing critical transitions in support and propulsion, consistent with the inverted pendulum model of terrestrial locomotion [9,13,15]. Sagittal-plane videos (30–120 fps) were digitized using Tracker software (version 6.1.6, Open Source Physics, Davidson College, 405 N. Main Street, Davidson, NC, USA, 2024). Anatomical landmarks (shoulder, elbow, wrist; hip, knee, ankle) were tracked frame by frame following species-specific skeletal models. This method has demonstrated high criterion validity and inter-observer reliability (ICC_3,1_ > 0.88; SEM < 13°) in mammalian gait studies [24].

Three functional metrics were calculated:

1. Joint Angular Excursion (JAE): defined as the difference between maximum and minimum joint angles during stance.
2. Total Angular Excursion (TAE): representing limb-level angular displacement [25].
3. Angular Efficiency Index (AEI): calculated as the ratio of TAE to the sum of JAE across limb joints [10,24,25]

$$AEI=\frac{TAE}{\sum JAE}\times 100$$

Higher AEI values indicate greater mechanical efficiency, whereas lower values reflect increased joint recruitment for the same limb displacement. TAE and AEI values were analyzed across four biological factors (body mass, limb posture, top locomotor speed, and locomotor habit) to identify locomotor trends and refine functional interpretations for *C. munozi*.

### 2.6. Biological Factors and Functional Estimation

Osteological indicators, functional indices, and stance-phase angular excursion values were analyzed in relation to four biological factors influencing locomotor performance [26,27]. Body mass was categorized as small (<1 kg), medium (1–29 kg), large (30–100 kg), or very large (>100 kg) [28,29,30]. These categories were complemented by body mass estimates derived from astragalar width using the predictive equation [12] (see Supplementary File S1):

$$BM=e^{\left(2.789\cdot \ln\left(Li1\right)+2.078\right)}\times 1.030$$

where BM is body mass (g) and Li1 is the transverse width of the tibial trochlea (mm).

Limb posture was classified as plantigrade, digitigrade, or unguligrade, based on astragalar morphology [6]. Top speed was categorized as slow (<35 km/h), medium (35–50 km/h), or fast (>50 km/h) [28], and locomotor habit was defined as arboreal, cursorial, scansorial, or terrestrial according to anatomical and ecological traits [31,32].

For mesotheriids, stance-phase angular excursions (TAE, ∑JAE, AEI) were inferred by assigning *Caraguatypotherium munozi* and *Trachytherus spegazzinianus* to extant functional categories that were morphologically most plausible, based on their astragalar indices (TDI, NFP, TRAA, NRAA, CRAA). Each mesotheriid was positioned within the morphospaces of modern mammals sharing equivalent combinations of body mass, limb posture, and locomotor habit. This comparative framework allowed estimating functional ranges of angular excursion from biologically and morphologically analogous taxa, providing a robust basis for reconstructing mesotheriid stance-phase mechanics.

### 2.7. Statistical Analysis

Principal component analyses (PCA) were conducted on the Pearson correlation matrix of size-free astragalar variables. Standardized values obtained from the preprocessing step (Section 2.4) were used as inputs, with fossil scores calculated by projection after applying the same scaling parameters as for extant taxa.

PCA results are presented as morphospaces, where species are positioned by their PC scores and extant groups are color-coded according to biological factors (body mass, posture, top speed, locomotor habit). This visualization facilitates the identification of functional clusters and overlap among categories.

The relative placement of *Caraguatypotherium munozi* and *Trachytherus spegazzinianus* with respect to extant clusters—together with PC loadings—was then used to infer dominant stance-phase biomechanical traits. Because PCA axes are sign-indeterminate, axis orientation was standardized across all panels for consistency.

Specimen-level PC scores, eigenvalues, loadings, and plotting information are provided in Supplementary File S2, ensuring full transparency and reproducibility.

Total Angular Excursion (TAE) and Angular Efficiency Index (AEI) were regressed against body mass, limb posture, and locomotor habit categories to evaluate potential scaling relationships and functional trade-offs among extant mammals.

Expected stance-phase angular excursion ranges for *C. munozi* † and *T. spegazzinianus* † were estimated using a conservative intersection approach. For each biological factor (body mass, limb posture, top locomotor speed, and locomotor habit), mean ± SD intervals of TAE, ∑JAE, and AEI were extracted from a comparative dataset of terrestrial mammal species [24]. The upper bound of the plausible range was defined as the lowest upper limit across factors, whereas the lower bound was defined as the highest lower limit, yielding a consensus range constrained by overlapping intervals. This approach reduces overestimation by simultaneously integrating all biological factors.

All analyses and figures were generated in GraphPad Prism version 6.0 (GraphPad Software, LLC, San Diego, CA, USA; 2012). Principal component analyses were run in R version 4.4.1 (R Foundation for Statistical Computing, Vienna, Austria; 2024) using custom scripts within RStudio 2024.04 (“Chocolate Cosmos”; Posit PBC, Boston, MA, USA). Minor graphical refinements were assisted by ChatGPT (OpenAI, GPT-4, San Francisco, CA, USA; 2024). All procedures complied with Chilean research regulations (Letter No. 1355/2023).

## 3. Results

### 3.1. Osteological Patterns of the Astragalus

**Figure 3:**
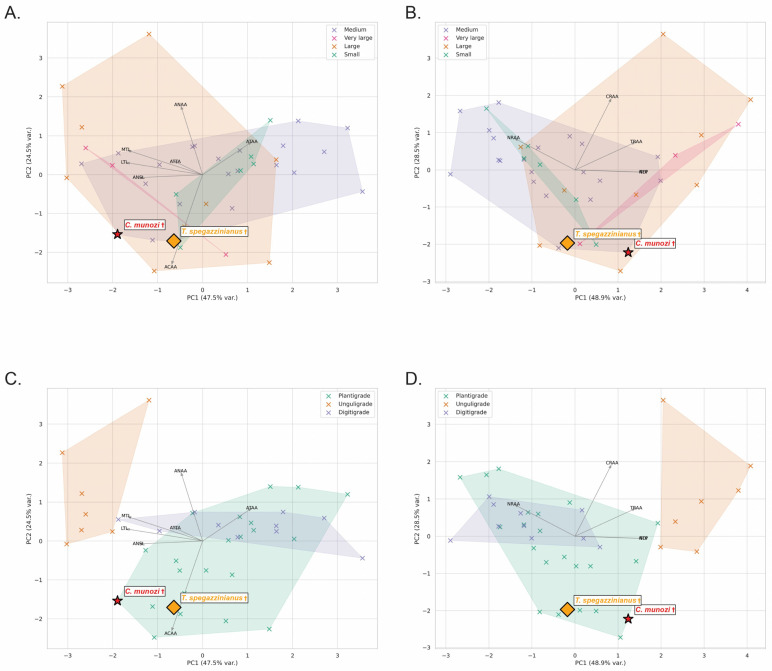
Principal component analyses (PCA) of astragalar variables in terrestrial mammals: (**A**,**C**) normalized osteological variables; (**B**,**D**) functional variables (biomechanical ratios). (**A**,**B**) Morphospaces grouped by body mass categories: Small (green), Medium (blue), Large (orange), and Very large (pink). (**C**,**D**) Morphospaces grouped by locomotor posture: Plantigrade (green), Digitigrade (blue), and Unguligrade (orange). The first two principal components explain 47.5% and 24.5% of the variance in the osteological dataset (**A**,**C**), and 48.9% and 28.5% in the functional dataset (**B**,**D**). Vectors indicate variable loadings. The placement of *Caraguatypotherium munozi* (red star) and *Trachytherus spegazzinianus* (yellow diamond) is highlighted as distinct, unassigned symbols. Inputs are size-free variables (normalized ratios, relative areas, and ATTA, °), z-standardized using extant means and standard deviations. PCA was computed on the correlation matrix. Because PCA axes are mathematically sign-indeterminate, axis orientation was standardized across panels for direct comparison. Species-level scores and plotting details are provided in Supplementary File S2. † indicates extinct species.

**Figure 4:**
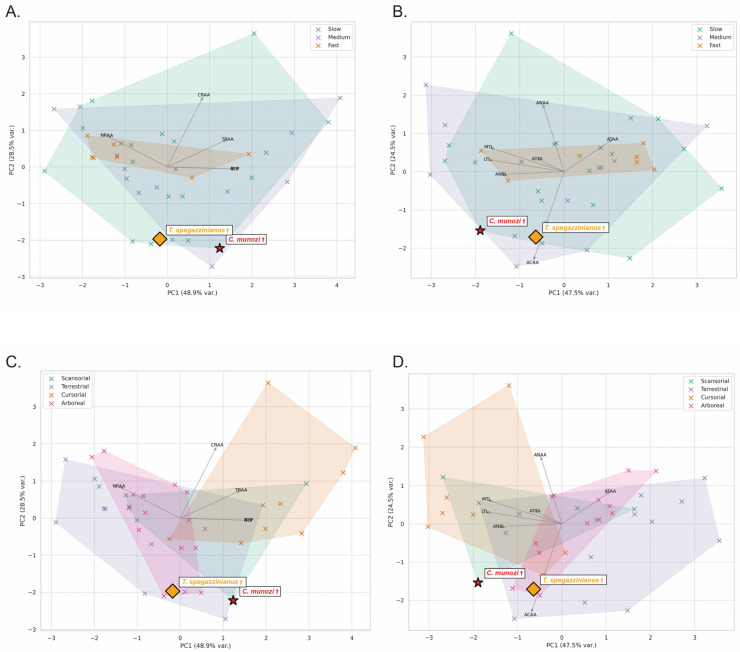
Principal component analyses (PCA) of astragalar variables in terrestrial mammals: (**A**,**C**) normalized osteological variables; (**B**,**D**) functional variables (biomechanical ratios). (**A**,**B**) Morphospaces grouped by locomotor speed categories: Slow (green), Medium (blue), and Fast (orange). (**C**,**D**) Morphospaces grouped by locomotor habit: Scansorial (green), Terrestrial (blue), Cursorial (orange), and Arboreal (pink). The first two principal components explain 47.5% and 24.5% of the variance in the osteological dataset (**A**,**C**), and 48.9% and 28.5% in the functional dataset (**B**,**D**). Vectors indicate variable loadings. The placement of *Caraguatypotherium munozi* (red star) and *Trachytherus spegazzinianus* (yellow diamond) is highlighted as distinct, unassigned symbols. Inputs are size-free variables (normalized ratios, relative areas, and ATTA, °), z-standardized using extant means and standard deviations. PCA was computed on the correlation matrix. Because PCA axes are mathematically sign-indeterminate, axis orientation was standardized across panels for direct comparison. Species-level scores and plotting details are provided in Supplementary File S2. † indicates extinct species.

The osteological dataset of 38 extant mammals revealed consistent trends in astragalar morphology across biological factors (Table 1; Figure 3A,C and Figure 4A,C). Body mass strongly influenced trochlear and articular dimensions: unguligrade large and very large mammals exhibited the highest mean lateral and medial trochlear lengths and tibial articular areas (e.g., LTL > 20 mm; ATAA > 200 mm^2^), reflecting increased weight-bearing demands. In contrast, small plantigrades presented reduced trochlear dimensions (e.g., LTL ≈ 7.5 mm; ATAA < 60 mm^2^), yet proportionally larger navicular and calcaneal articular areas relative to body size, suggesting enhanced multidirectional stability.

Posture categories formed well-separated clusters. Unguligrades displayed deep, narrow trochleae (high TDI) and robust trochlear widths (AMWT > 20 mm), whereas plantigrades exhibited broader articular surfaces (higher ANAA and ACAA), consistent with functional demands for mediolateral stability during slower locomotion.

Top speed correlated with trochlear depth and articular proportions. Fast-moving taxa (e.g., cursorial digitigrades) showed higher TDI and smaller navicular facets (lower ANSL and ANAA), indicating increased parasagittal constraint. Conversely, slow-moving plantigrades exhibited shallow trochleae (low TDI) and expanded navicular and calcaneal areas, favoring multidirectional mobility.

Locomotor habits also explained significant variation: cursorial mammals clustered in morphospaces defined by high PC1 scores (size-related variables: ATAA, ACAA, AMWT), whereas arboreal and scansorial plantigrades shifted toward higher PC2 values, associated with increased navicular surface length (ANSL) and broader trochlear proportions. Semifossorial plantigrades occupied an intermediate range, with moderate trochlear widths and enlarged calcaneal articular areas.

Principal Component Analysis (PCA) of log-transformed variables explained 72% of total variance (PC1 = 47.5%, PC2 = 24.5%). PC1 primarily reflected size and weight-bearing variables (ATAA, ACAA, AMWT), while PC2 captured shape-functional contrasts, separating species by trochlear proportions (LTL, MTL) and navicular surface length (ANSL).

The two mesotheriid species plotted within the plantigrade morphospace but with distinct tendencies. *C. munozi* † was displaced toward higher navicular proportions and lower trochlear depth (lower PC2 scores), aligning closer to scansorial plantigrades (e.g., *Potos flavus*, *Eulemur fulvus*). In contrast, *T. spegazzinianus* † occupied a more central plantigrade position, resembling terrestrial–semifossorial taxa (*Vombatus ursinus*, *Taxidea taxus*), consistent with its inferred stability-biased locomotion (see Supplementary File S2).

### 3.2. Results for Astragalar Functional Indices

Functional indices showed clear trends across all biological factors (Table 2; Figure 3A–D). Body mass strongly influenced astragalar shape, with larger and very large mammals exhibiting higher TDI (2.02 ± 0.59 and 2.01 ± 0.28, respectively) and markedly greater NFP values (up to 14.23 ± 8.55), consistent with increased parasagittal stability and cursorial tendencies. Postural categories were well separated: unguligrades displayed the highest TDI (2.44 ± 0.28) and NFP (13.05 ± 5.74), while plantigrades maintained lower TDI (1.59 ± 0.27) and moderate NFP (4.01 ± 4.27), reflecting greater mediolateral mobility.

Top locomotor speed also influenced astragalar function. Medium-speed mammals (35–50 km/h) showed slightly higher TDI (1.90 ± 0.57) and NFP (7.25 ± 5.36) than slow or fast taxa, suggesting that moderate speeds may favor a balance between mobility and stability. Fast mammals (>50 km/h), although few in the sample, clustered with digitigrades, characterized by higher TDI (1.75 ± 0.44) and TRAA (0.61 ± 0.08), indicative of increased parasagittal constraint.

Locomotor habit exerted a marked effect on articular proportions: cursorial mammals showed the highest TDI (2.21 ± 0.47) and NFP (12.86 ± 5.84), whereas arboreal and terrestrial plantigrades had lower TDI (<1.7) and NFP (~4.20 ± 3.74), consistent with more compliant, multidirectional ankle use. Scansorial taxa occupied an intermediate position, with moderately high TDI (1.88 ± 0.43) and TRAA (0.58 ± 0.10), balancing stability and mobility.

PCA based on functional indices explained 81.4% of the variance (PC1 = 62.7%; PC2 = 18.7%). PC1 was dominated by TDI and NFP, separating cursorial unguligrades from plantigrades and scansorials (i.e., arboreals in Supplementary File S2), while PC2 reflected relative articular areas (TRAA, NRAA, CRAA).

The two mesotheriids plotted within the plantigrade morphospace but in distinct positions. *C. munozi* † aligned closer to scansorial plantigrades, showing low NFP (1.61) and moderate TRAA (0.52), suggesting greater mediolateral mobility. *T. spegazzinianus* †, in contrast, clustered with terrestrial plantigrades, characterized by slightly higher TDI (1.94) and comparable TRAA (0.53), consistent with a more stability-biased, semifossorial habit (see Supplementary File S2).

### 3.3. Plausible Biological Factors of Mesotheriidae

Integration of osteological and functional evidence supports distinct locomotor profiles for the two mesotheriids. *Caraguatypotherium munozi* † exhibited astragalar traits consistent with a plantigrade, medium-to-large-bodied mammal (~17–35 kg, estimated using Tsubamoto’s formula), characterized by slow locomotion and a terrestrial–scansorial strategy. Its relatively low Navicular Facet Proportion (NFP) and reduced Trochlear Depth Index (TDI) indicate enhanced mediolateral mobility combined with stable weight support, resembling scansorial plantigrades such as *Potos flavus* or *Eulemur fulvus*.

In contrast, *Trachytherus spegazzinianus* † showed astragalar proportions typical of a plantigrade, medium-bodied mammal (~13–27 kg, estimated using Tsubamoto’s formula), adapted for slow terrestrial locomotion with semifossorial tendencies. Its higher TDI and NFP, together with more centrally distributed osteological proportions, align closely with terrestrial–semifossorial analogs such as *Vombatus ursinus* and *Taxidea taxus*.

These results suggest that, despite their phylogenetic proximity, the two mesotheriids likely occupied different functional niches: *C. munozi* † exhibited a morphofunctional spectrum biased toward greater scansorial versatility, whereas *T. spegazzinianus* † retained a more terrestrial–semifossorial profile.

Based on these biologically plausible categories, expected stance-phase kinematic ranges were estimated by intersecting the factor-specific intervals for Total Angular Excursion (TAE) and Angular Efficiency Index (AEI). Mechanical efficiency was defined as the ratio between total limb angular displacement (TAE) and the summed joint angular excursion (∑JAE) during the stance phase, following the approach proposed by Schmidt (2005) [25]. This intersection method provided a conservative estimate of functional ranges, constrained by the overlapping intervals of all relevant biological factors.

### 3.4. Applying the Mammalian Kinematic Dataset: Estimated Angular Excursion Ranges

**Figure 5:**
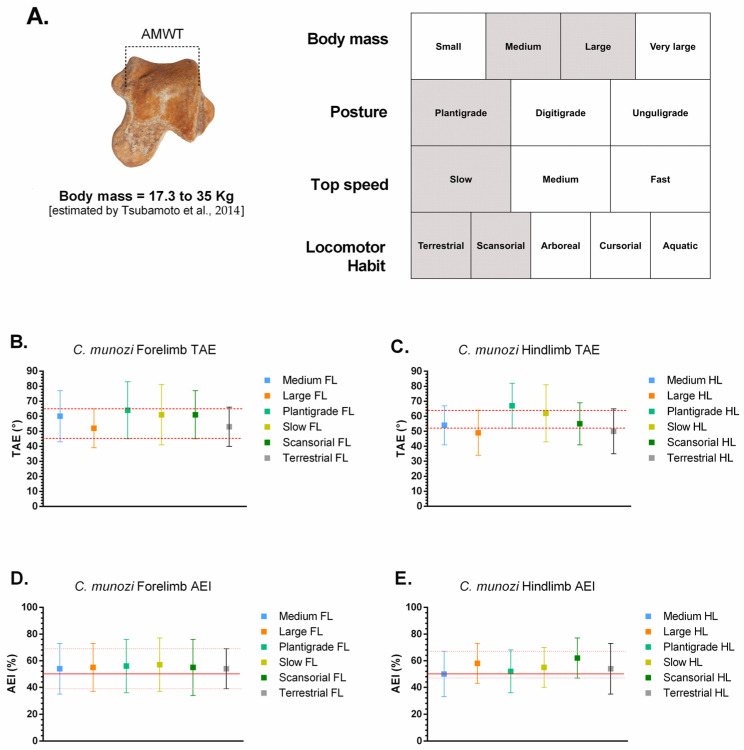
Estimated stance-phase functional ranges for *Caraguatypotherium munozi* based on extant analogs. (**A**) Biological categories inferred from astragalar morphology: medium-to-large body mass, plantigrade posture, slow-to-medium top speed, and terrestrial–scansorial locomotor habit. Body mass range estimated using Tsubamoto et al. (2014) [12]. (**B**) Total Angular Excursion (TAE) of the forelimb (°). (**C**) TAE of the hindlimb (°). (**D**) Angular Efficiency Index (AEI) of the forelimb (%). (**E**) AEI of the hindlimb (%). Dots represent mean values, and vertical lines indicate ±1 SD for each biological category (Medium, Large, Plantigrade, Slow, Scansorial, and Terrestrial). The red dashed lines show the consensus range derived from the intersection of all relevant biological factors, representing the most plausible functional range for *C. munozi* during stance-phase locomotion.

**Figure 6:**
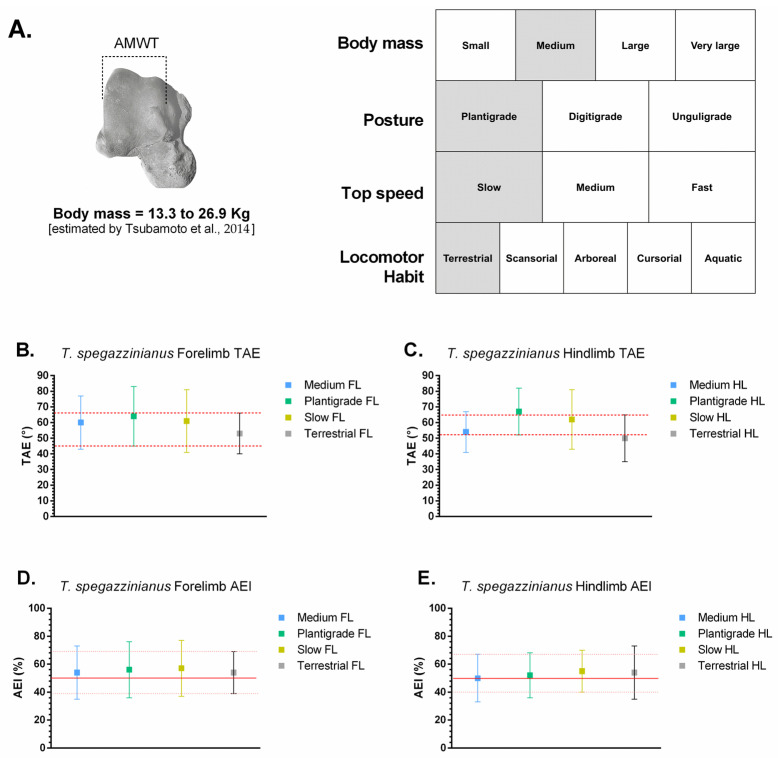
Estimated stance-phase functional ranges for *Trachytherus spegazzinianus* based on extant analogs. (**A**) Biological categories inferred from astragalar morphology: medium body mass, plantigrade posture, slow top speed, and terrestrial locomotor habit with semifossorial tendencies. Body mass range estimated using Tsubamoto et al. (2014) [12]. (**B**) Total Angular Excursion (TAE) of the forelimb (°). (**C**) TAE of the hindlimb (°). (**D**) Angular Efficiency Index (AEI) of the forelimb (%). (**E**) AEI of the hindlimb (%). Dots represent mean values, and vertical lines indicate ±1 SD for each biological category (Medium, Plantigrade, Slow, and Terrestrial). The red dashed lines indicate the consensus range derived from the intersection of all relevant biological factors, representing the most plausible functional range for *T. spegazzinianus* during stance-phase locomotion.

The stance-phase kinematic ranges estimated for *Caraguatypotherium munozi* † and *Trachytherus spegazzinianus* † were derived by anchoring their most plausible biological categories (body mass, posture, top locomotor speed, and locomotor habit) to the comparative angular excursion dataset of extant mammals (Table 3; Figure 5 and Figure 6; [24]). Despite occupying distinct morphospaces based on osteological and functional indicators, both species exhibited largely overlapping kinematic ranges, reflecting the functional conservatism of the plantigrade module.

In Figure 5, *C. munozi* † plots within a transitory plantigrade morphospace, closer to scansorial analogs, whereas In Figure 6, *T. spegazzinianus* † is restricted to a more stable terrestrial–semifossorial region. The results shows the superposed functional ranges of TAE and AEI, where both species share broadly similar values, indicating comparable stance-phase mechanics despite their differing ecomorphological profiles.

For *C. munozi* † (plantigrade, medium-to-large-bodied, slow-moving, terrestrial–scansorial):

- Forelimb TAE: ~45–65°; AEI: ~40–65%.
- Hindlimb TAE: ~52–64°; AEI: ~44–64%.

For *T. spegazzinianus* † (plantigrade, medium-bodied, slow, terrestrial–semifossorial):

- Forelimb TAE: ~45–66°; AEI: ~39–69%.
- Hindlimb TAE: ~52–65°; AEI: ~40–67%.

These results suggest that, although *C. munozi* † possessed a broader spectrum of biological capabilities consistent with a scansorial tendency, both mesotheriids shared a functionally conservative plantigrade kinematic module during the stance phase of walking.

## 4. Discussion

The astragalus constitutes a key biomechanical hinge between the autopodium and the proximal limb, acting as the primary modulator of force transfer to the substrate during stance [6,11]. Beyond transmitting vertical and shear ground reaction forces, it redistributes these forces through the calcaneal complex, modulating stability and mobility according to articular morphology [18,33]. Trochlear depth and articular surface orientation encapsulate evolutionary trade-offs: a deep, pulley-like trochlea constrains movement to the parasagittal plane, maximizing energy transfer and stability in cursorial species, whereas shallower trochleae and dorsoplantarly expanded navicular and calcaneal facets increase mediolateral mobility, typical of plantigrade and scansorial taxa [1,11]. Recent analyses have further emphasized that astragalar shape reflects a complex interplay of allometric, phylogenetic, and ecological factors, with the degree of development of articular and ligamentous regions directly influencing ankle mobility and locomotor performance [34].

These results partially confirm the proposed functional framework in mesotheriids, but with an important nuance: despite distinct osteological morphospaces, both species exhibited largely overlapping stance-phase kinematics. *Caraguatypotherium munozi* † showed a deeper trochlea, moderately expanded navicular facets, and slightly higher TAE and AEI, consistent with a stable but mechanically compliant ankle favoring terrestrial–scansorial locomotion. Conversely, *Trachytherus spegazzinianus* † displayed a shallower trochlea and narrower articular surfaces, aligning with a more stability-biased, semifossorial profile. These differences place both species in separate regions of the plantigrade morphospace, with *C. munozi* † closer to scansorial analogs and *T. spegazzinianus* † nearer to semifossorial plantigrades (e.g., *Vombatus ursinus*).

However, when compared against the extant kinematic dataset, their TAE and AEI ranges overlapped substantially, suggesting that stance-phase mechanics in plantigrade mesotheriids were more conservative than their osteological disparity might imply. This functional conservatism highlights the astragalus as a biomechanical “link” whose overall locomotor role can remain stable even when subtle morphological shifts allow ecological diversification. Thus, while the two mesotheriids likely occupied distinct ecological niches, they did so within a shared plantigrade functional module, reflecting the evolutionary plasticity of Mesotheriidae constrained by a common weight-bearing strategy.

### 4.1. Functional Diversity Within Mesotheriidae

The traditional interpretation of mesotheriids as generalized terrestrial diggers [17] is challenged by the present findings, which reveal distinct locomotor profiles despite similar functional modules. Both *Caraguatypotherium munozi* and *Trachytherus spegazzinianus* share a conservative plantigrade kinematic module, as indicated by their overlapping stance-phase ranges of Total Angular Excursion (TAE) and Angular Efficiency Index (AEI), Figure 5 and Figure 6. This overlap suggests that their ankle joints operated within similar mechanical limits during walking. However, the osteological and functional morphospaces reveal clear ecomorphological divergence: *C. munozi* exhibits lower Trochlear Depth Index (TDI), moderate navicular expansion, and slightly higher hindlimb AEI values approaching the upper limits of plantigrade efficiency, suggesting more effective recruitment of available mobility per step and enhanced maneuverability on irregular substrates. In contrast, *T. spegazzinianus* maintains higher TDI and more centralized astragalar proportions, consistent with a stability-biased, semifossorial profile optimized for resisting substrate forces during digging.

This pattern aligns with evidence from *Plesiotypotherium*, where subtle variations in articular proportions have been shown to translate into significant functional shifts, even among scratch-diggers [17,18]. Differences in articular surface orientation, even within closely related taxa, strongly modulate mediolateral ankle mobility [18], while astragalar variability has been interpreted as reflecting adaptive responses rather than neutral variation, supporting the ecological significance of these differences [35].

Finally, the broader context provided by early notoungulates corroborates this interpretation. Evidence indicates that taxa such as *Notostylops* and *Notopithecus* exhibited functional plasticity despite sharing generalist postcranial morphologies [27]. Together, these findings suggest that the mesotheriid astragalus retained a phylogenetically conservative mechanical framework while allowing adaptive modulation of stance-phase performance, thus enabling ecological diversification within Mesotheriidae.

### 4.2. Morphospace Patterns and Comparative Functional Morphology

The Principal Component Analysis (PCA) of both osteological variables and functional indices reinforces the evidence for ecological differentiation between the two mesotheriids. Although *Caraguatypotherium munozi* and *Trachytherus spegazzinianus* cluster within the plantigrade morphospace of extant terrestrial mammals—consistent with previously inferred fossorial habits [16,17]—their distribution along the mobility–stability gradient reveals contrasting functional tendencies. *C. munozi* occupies a more transitional position, overlapping with scansorial plantigrades such as Potos flavus and Eulemur fulvus, whereas *T. spegazzinianus* remains tightly associated with terrestrial–semifossorial analogs, including *Vombatus ursinus* and *Taxidea taxus* (Supplementary File S2).

Body mass provides an additional axis of inference that refines this functional reading of the morphospaces. In *C. munozi*, positions near the margins of extant envelopes across PCAs (Figure 3A,B) permit alternative placements spanning the medium–large continuum. When combined with predictive equations, however, this ambiguity is reduced: the Li1 (Tsubamoto) estimator yields consistently medium-to-large values (Figure 5 and Figure 6; Supplementary File S1), whereas *T. spegazzinianus* aligns more stably with a medium body-mass class across both morphospaces and predictive outputs. These assignments are congruent with phylogenetic evidence on mesotheriid size evolution [16] and with paleobiological reconstructions for related notoungulates [20]. Taken together, the PCA morphospaces (size-free osteological variables and functional indices), the mechanical estimator (Tsubamoto), and comparative phylogenetic/paleobiological information converge on a coherent picture: *C. munozi* was medium–large, while *T. spegazzinianus* was medium.

This spatial segregation in morphospace highlights the astragalus’s sensitivity to ecological pressures and supports the value of integrating osteological and functional indices to reconstruct morphofunctional spaces. In line with the form–function complex framework [1,5], such divergence likely reflects differential environmental selective pressures acting on closely related taxa. Rather than being strictly fossorial generalists, mesotheriids appear to have diversified into distinct locomotor strategies within a shared plantigrade module, suggesting greater ecological and evolutionary plasticity than traditionally assumed. More broadly, this synthesis illustrates the ductility of morphofunctional spaces as heuristic devices: by integrating independent lines of evidence, robust paleobiological solutions can be developed [26].

### 4.3. Kinematic Morphofunctional Spaces and Novel Analytical Framework

Figure 5 and Figure 6 provide the first quantitative attempt to estimate stance-phase kinematic ranges in mesotheriids by integrating multiple biological factors, marking an important step in testing traditional paleobiological inferences about this group. By intersecting mean ± SD intervals of Total Angular Excursion (TAE) and Angular Efficiency Index (AEI) from extant taxa categorized by posture, body mass, locomotor habit, and top speed, this approach refines functional estimations beyond qualitative analogies. It demonstrates that, despite overall similarity in kinematic parameters, subtle functional divergences emerge when interpreted within a morphofunctional and ecological context.

*Caraguatypotherium munozi* †, identified as a plantigrade, medium-to-large-bodied (~17–35 kg), slow-moving terrestrial–scansorial species, *C. munozi* aligns with extant scansorial plantigrades (*Potos flavus*, *Eulemur fulvus*). Its estimated forelimb and hindlimb excursions (TAE ~45–65°; AEI ~40–65%) place it within a moderately mobile plantigrade morphofunctional space, suggesting enhanced mediolateral mobility and stability compared to typical terrestrial plantigrades. The slightly higher hindlimb AEI values, closer to the upper efficiency limits observed in plantigrade mammals, imply that *C. munozi* optimized the redistribution of angular displacement across joints rather than relying on greater absolute joint mobility. This efficient recruitment of available joint excursion could have provided maneuverability on irregular or heterogeneous substrates, supporting its transitional morphospace position and inferred ecological versatility.

*Trachytherus spegazzinianus* †, classified as a plantigrade, medium-bodied (~13–27 kg), slow terrestrial–semifossorial species, *T. spegazzinianus* shows greater affinity with terrestrial plantigrades such as *Vombatus ursinus* and *Taxidea taxus*. Its functional ranges (forelimb TAE ~45–66°; AEI ~39–69%) overlap extensively with those of *C. munozi*, but its morphospace position and slightly lower hindlimb AEI suggest a functional emphasis on stability. This stability-biased ankle configuration is consistent with semifossorial habits and resistance to substrate forces during digging.

The overlap in TAE and AEI between the two species highlights a functionally conservative plantigrade kinematic module during the stance phase. However, the subtle differences in efficiency and morphospace positioning likely reflect divergent ecological pressures related to their distinct temporal and geographic distributions. *C. munozi*’s scansorial tendencies appear to derive from how it modulated its available joint excursion—achieving higher mechanical efficiency with similar absolute ranges—whereas *T. spegazzinianus* prioritized stability within the same kinematic framework.

This interpretation suggests that functional diversity within Mesotheriidae may have arisen not primarily from differences in absolute joint mobility but from distinct strategies of biomechanical modulation within a shared plantigrade module. Such findings emphasize the ecological plasticity of mesotheriids and expand the understanding of their locomotor adaptations beyond the traditional view of generalized terrestrial diggers.

### 4.4. Functional Conservatism of the Astragalus Within Plantigrade Mesotheriids

Although the astragalus exhibits high morphological variability across modern mammals—reflecting divergent adaptations to cursorial, arboreal, or semifossorial habits [6,11]—evidence nevertheless points to strong functional conservatism in stance-phase kinematics among plantigrade taxa. This finding is consistent with the idea that, despite considerable variability in trochlear depth, articular facet proportions, and tarsal configurations, plantigrade astragali remain biomechanically constrained by the dual requirement of stability and multidirectional mobility during slow terrestrial locomotion [9,10].

Within Notoungulata, astragalar–tarsal arrangements (e.g., alternating, reversed alternating, and serial configurations) exhibit notable morphological plasticity that is not strictly constrained by phylogeny [35]. Instead, these variations appear to be functionally canalized, maintaining a weight-supporting and force-transmission module optimized for plantigrade locomotion.

The similar TAE and AEI ranges observed for *Caraguatypotherium munozi* and *Trachytherus spegazzinianus* exemplify this conservatism. Both mesotheriids occupy a morphofunctional space comparable to extant plantigrade and terrestrial mammals, suggesting that once a plantigrade locomotor module is established, functional divergence may remain constrained even among taxa with distinct ecological tendencies. This interpretation is consistent with observations indicating that tarsal variability in notoungulates seldom produces major functional shifts when the primary weight-supporting mechanics remain unchanged [35].

### 4.5. Ecomorphological Divergence Despite Functional Overlap

Despite this overarching functional conservatism, the PCA-derived morphospaces reveal ecomorphological differences not fully captured by TAE and AEI. *C. munozi* occupies a more transitional morphospace, associated with medium-to-large body mass, slow-to-medium top speed, and a terrestrial–scansorial habit, indicating greater ecological versatility and potential maneuverability on irregular substrates. In contrast, *T. spegazzinianus*, consistently classified as medium-sized, slow, and strictly terrestrial, is positioned closer to semifossorial specialists.

This pattern supports the hypothesis that functional similarity in gross kinematic parameters can mask ecologically relevant differences. Subtle shifts in astragalar facet proportions, head orientation, and the relative involvement of calcaneal and navicular facets can modulate how joint mobility is recruited without altering absolute angular ranges [11,35]. Such microecomorphological adjustments likely underlie the broader locomotor spectrum inferred for *Caraguatypotherium munozi*, enabling the exploitation of a wider range of substrates, whereas *Trachytherus spegazzinianus* remained functionally constrained to stability-biased terrestrial habits.

### 4.6. Evolutionary and Biomechanical Implications

These findings reinforce the concept of the astragalus as a biomechanical hinge—a structure that, while responsive to ecological pressures, remains evolutionarily conservative in its core role of weight support and force transmission [33]. The moderate astragalar variability observed among mesotheriids supports previous interpretations that tarsal elements in Typotheria exhibit strong functional integration [12], particularly in taxa maintaining a predominantly plantigrade stance. Such integration appears to have constrained kinematic diversification, favoring stability and energy-efficient locomotion over cursorial adaptations.

Within Mesotheriidae, this pattern suggests an evolutionary scenario in which multiple ecological strategies—ranging from fossorial to semi-cursorial behaviors—evolved within a shared plantigrade functional module. Morphometric indices, such as tibial, femoral, and astragalar robustness, indicate that typotheres, and especially mesotheriids, prioritized joint stability and force generation over mobility [12], consistent with the reduced trochlear depth and robust astragalar–calcaneal articulations documented here. Similar conservatism has been noted in other notoungulates, where astragalar–tarsal configurations show significant morphological plasticity but remain functionally canalized to maintain plantigrade weight-bearing efficiency [35]. This aligns with evidence from early notoungulates, which displayed functional plasticity despite generalist postcranial morphologies [27], supporting the view that subtle structural adjustments, rather than major kinematic changes, drove ecological diversification.

Beyond osteological proxies, ichnological records provide additional insight into the locomotor ecology of notoungulates. Extensive networks of large fossil burrows (paleoburrows) from Argentina, Uruguay, and Brazil have been attributed to Cenozoic mammals, including typotheres and toxodontids, and are consistent with semifossorial behaviors [36,37]. While no ichnofossils have been directly associated with Mesotheriidae, the presence of such structures within other notoungulate clades suggests that fossoriality was a recurrent ecological option within the order. The astragalar morphology of *Caraguatypotherium munozi* and *Trachytherus spegazzinianus* documented in this study—characterized by enhanced joint stability, robust astragalar–calcaneal articulations, and limited trochlear depth—is biomechanically consistent with the demands of scratch-digging or semifossorial activity. Importantly, this morphology coexists with a conservative plantigrade framework optimized for weight-bearing stability and versatile substrate interaction, indicating that mesotheriids were not obligate diggers but likely engaged in a spectrum of substrate-related behaviors. This convergence between osteological proxies and ichnological evidence supports an ecological scenario in which mesotheriids combined generalized plantigrade locomotion with facultative substrate modification, paralleling strategies inferred for other South American ungulates during the Miocene.

Comparative evidence indicates that semifossorial and cursorial adaptations were not exclusive to mesotheriids. *Protypotherium* shows a mosaic of cursorial and fossorial traits in the postcranium, occupying an intermediate morphospace between runners and scratch-diggers [38]. Early notoungulates likewise exhibit postcranial diversity consistent with variable foot postures and locomotor strategies [39], and exceptionally preserved skeletons such as *Thomashuxleya externa* reveal generalized but functionally versatile limb configurations compatible with plantigrade support [40]. Cranial endocast data add an independent axis of comparison: variation in the bony labyrinth across notoungulates captures both phylogenetic signal and aspects of locomotor performance [41], reinforcing the view that ecological disparity arose within a broadly conserved locomotor framework rather than via radical departures from plantigrady. Functional analyses of middle Eocene typotheres (*Notostylops*, *Notopithecus*) similarly document differentiated limb function within a shared structural template [27]. Taken together, these patterns underscore that mesotheriid astragalar adaptations are best interpreted within a broader notoungulate continuum in which locomotor diversity emerged through differential emphasis on robustness, stability, and mobility rather than wholesale reorganization of stance-phase mechanics.

Histological evidence provides a convergent line of support. Paleohistology of *Caraguatypotherium munozi* indicates slow bone deposition, elevated cortical thickness, and signatures of sustained loading regimes [42], consistent with reinforced astragalar–calcaneal articulations and reduced trochlear depth documented here. Independent forelimb evidence from *C. munozi*—including wrist-powered digging capabilities—further supports enhanced force transmission and joint stability during stance [26]. The convergence of articular morphology, histology, and forelimb function argues that mesotheriids optimized weight-bearing efficiency within a conservative plantigrade module, while ecological disparity was achieved through fine-scale modulation of musculoskeletal function rather than major kinematic shifts.

Finally, the broader evolutionary context proposed for endemic South American ungulates suggests that, despite remarkable tarsal variability, notoungulates retained a phylogenetically conservative astragalar framework optimized for plantigrade locomotion [43]. This supports the hypothesis that ecological diversification within Mesotheriidae was achieved not through major reorganization of stance-phase mechanics, but through subtle adjustments in limb robustness, joint morphology, and muscle recruitment. In this sense, mesotheriids exemplify how morphological conservatism at the joint level can coexist with ecological and functional disparity at the clade level.

### 4.7. Limitations and Future Directions

Although the present inferences are robust, they rely primarily on extrapolations from osteological proxies and extant analogs. Soft tissue architecture—particularly ligaments, tendons, and joint capsules—which could substantially influence joint mobility and mechanical efficiency, remains unknown. Moreover, the estimated functional ranges (TAE, ∑JAE, AEI) are derived from modern taxa with similar biological traits [24], introducing some degree of uncertainty. Expanding mesotheriid samples and incorporating additional postcranial elements (e.g., calcaneus, phalanges) will be essential to refine these interpretations.

Another limitation concerns the inclusion of six domestic taxa in the extant comparative dataset. Domestication is known to alter musculoskeletal development and functional morphology, potentially decoupling anatomy from the ecological constraints observed in wild relatives [44,45]. Although the present analyses indicate that body mass exerts a stronger influence on astragalar morphology than domestic versus wild status, future studies should preferentially incorporate wild representatives to minimize potential biases introduced by artificial selection and husbandry. Addressing this issue will require broader, taxonomically balanced datasets to refine functional inferences in extinct taxa.

Recent advances in morpho-functional analysis using Procrustes Superimposition by Static Reference (PSSR) [46] highlight the evolutionary conservatism of articular surfaces and their suitability as stable biomechanical references. Applying this framework to mesotheriid astragali would allow disentangling functional (adaptive) from phylogenetic shape variation, refining morphospace-based inferences. Additionally, subarea-level analyses of articular surfaces could reveal microstructural integration patterns and localized stress adaptations not captured by traditional linear or areal proxies.

Advanced techniques such as finite element analysis (FEA) and force-distribution modeling applied to high-resolution CT or surface scans of mesotheriid astragali could elucidate stress pathways and loading regimes during stance [47,48]. Similarly, the incorporation of paleohistological data from long bones—already available for *C. munozi*—ref. [42] may provide independent evidence on growth patterns, mechanical loading, and potential locomotor specializations. Integrating mechanotransduction principles could further clarify how morphological variation translated into functional performance.

Future research should also aim to develop a comprehensive morphofunctional model, tracing the continuum from shape to function following established conceptual frameworks [1,2,5]. A schematic morphofunctional pathway linking morphological integration, kinematic performance, and ecological role could provide a unifying interpretative model for mesotheriid locomotion.

Finally, broader comparative datasets, including 3D geometric morphometrics of astragalar articular surfaces and biomechanical simulations, are necessary to test the functional ranges inferred here. Whether the observed functional disparity is representative of Mesotheriidae as a whole or reflects lineage-specific adaptations remains an open question. Complementary evidence from ichnological records and paleohistological studies could further elucidate habitual locomotor behaviors and substrate interactions in these extinct mammals.

## 5. Conclusions

This study underscores the critical role of the astragalus as a biomechanical hinge between the autopodium and the proximal limb, serving as the primary interface for transmitting ground reaction forces and muscular loads during stance-phase locomotion. Its osteological and functional attributes effectively capture the trade-offs between stability and mobility, confirming its reliability as a proxy for reconstructing posture, body mass, and locomotor ecology in extinct mammals.

Contrary to the long-standing view of mesotheriids as functionally homogeneous terrestrial diggers, the evidence demonstrates clear ecomorphological disparity within the clade. *Caraguatypotherium munozi* occupied a plantigrade, terrestrial–scansorial functional space characterized by moderate joint mobility and a morphospace position associated with medium-to-large body mass, slow locomotor speeds, and scansorial tendencies. In contrast, *Trachytherus spegazzinianus* exhibited a plantigrade, terrestrial–semifossorial profile consistent with a stability-biased locomotor strategy.

Despite these ecological differences, both species showed broadly overlapping stance-phase kinematic ranges. Estimated Total Angular Excursion (TAE) and Angular Efficiency Index (AEI) fell within similar intervals for forelimbs and hindlimbs, reflecting a shared plantigrade locomotor module strongly constrained by stability and energy-efficient weight support. This functional conservatism mirrors patterns in extant plantigrade mammals, where subtle modifications in astragalar morphology enable ecological diversification without major changes in gross kinematic performance.

Principal component analyses and morphofunctional reconstructions therefore suggest that mesotheriids exploited distinct ecological niches through minor morphological adjustments within a conserved locomotor framework. This highlights an evolutionary and ecological plasticity within Mesotheriidae that has been previously underappreciated, emphasizing the importance of integrating osteological proxies with kinematic datasets to disentangle ecological specialization from underlying functional constraints.

Overall, this study provides the first quantitative estimations of stance-phase mechanics in mesotheriids, showing that ecological divergence could emerge within a conserved plantigrade locomotor module. By integrating osteological proxies, kinematic analogs, and comparative evidence, the results highlight that subtle morphological adjustments—rather than major departures in joint function—likely underpinned ecological disparity in this clade. These estimations must be understood as context-specific, reflecting stance-phase locomotor mechanics within a plantigrade framework rather than absolute reconstructions of full gait dynamics. Future work incorporating broader comparative datasets and advanced biomechanical modeling will be essential to test whether these estimated patterns reflect a general trend across Mesotheriidae or a lineage-specific evolutionary trajectory.

## Figures and Tables

**Table 1:** Summary of astragalar osteological measurements across body mass, posture, top speed, and locomotor habit.

Biological Factors		LTL (mm)	MTL (mm)	ATAA (mm^2^)	ANAA (mm^2^)	AMWT (mm)	ANSL (mm)	ATTA (angle)	ACAA (mm^2^)
Body mass	Small (*n* = 6)	7.5 ± 8.0	6.5 ± 6.7	56 ± 106	11 ± 19	6.3 ± 5.8	5.1 ± 4.6	147 ± 7	54 ± 115
	Medium (*n* = 20)	13.3 ± 9.1	12.2 ± 7.6	157 ± 246	49 ± 122	11.6 ± 6.8	9.3 ± 7.0	145 ± 16	95 ± 208
	Large (*n* = 9)	33.4 ± 6.5	29.6 ± 6.2	558 ± 269	204 ± 129	25.0 ± 6.2	20.0 ± 5.8	161 ± 12	460 ± 357
	Very large (*n* = 3)	58.9 ± 14	53.4 ± 13.1	1241 ± 208	617 ± 507	42.4 ± 5.0	38.2 ± 13.7	157 ± 19	1152 ± 257
Posture	Plantigrade (*n* = 21)	16.1 ± 13.8	14.2 ± 11.5	295 ± 416	83.41 ± 155.62	14.58 ± 11.92	11.35 ± 9.97	145.27 ± 13.69	272.67 ± 414.73
	Digitigrade (*n* = 10)	15.0 ± 7.9	13.5 ± 6.7	181 ± 171	32 ± 28	13 ± 6.7	9.7 ± 4.6	146 ± 10	74 ± 65
	Unguligrade (*n* = 7)	43.0 ± 18.1	39.6 ± 16.0	606 ± 478	382 ± 372	26.39 ± 12.95	25.3 ± 14.6	171 ± 6	480 ± 487
Top speed	Slow (*n* = 20)	16.78 ± 19.06	15.21 ± 17.24	222.59 ± 388.37	113.76 ± 275.14	13.25 ± 12.95	11.17 ± 12.74	147.91 ± 16.03	182.01 ± 363.55
	Medium (*n* = 11)	26.6 ± 14.9	24.0 ± 13.1	458 ± 421	150 ± 148	20.22 ± 10.69	16.3 ± 9.2	159 ± 14	417 ± 450
	Fast (*n* = 7)	22.7 ± 11.2	20.2 ± 8.4	392 ± 365	116 ± 200	19.04 ± 8.14	15.7 ± 9.4	143 ± 8	229 ± 331
Locomotor habit	Arboreal (*n* = 13)	7.7 ± 5.4	7.0 ± 4.5	52 ± 70	13 ± 14	6.65 ± 4.07	5.5 ± 3.2	144 ± 13	39 ± 76
	Cursorial (*n* = 8)	42.18 ± 16.97	37.82 ± 15.59	734.14 ± 462.72	393.49 ± 347.61	28.56 ± 11.99	27.14 ± 13.3	166.74 ± 7.78	625.5 ± 513.21
	Scansorial (*n* = 4)	22.4 ± 9.8	18.8 ± 8.8	294 ± 195	73 ± 84	16.67 ± 7.57	12.9 ± 4.6	155 ± 14	174 ± 119
	Terrestrial (*n* = 13)	20.18 ± 12.78	18.32 ± 10.85	350.59 ± 391.68	82.94 ± 149.08	18.39 ± 10.36	13.23 ± 8.7	144.19 ± 13.05	288.56 ± 394.48
Mesotheridae †	*C. munozi* † (*n* = 1)	24.6	16.7	345.0	19.0	12.8	11.8	152.0	301.3
	*T. spegazzinianus* † (*n* = 1)	25.5	19.2	414.0	27.0	18.1	18.0	144.5	341.0

Values represent mean ± standard deviation (*n* = number of species). Mean ± standard deviation (*n* = number of species) of absolute osteological measurements of the astragalus, grouped by body mass, limb posture, top speed, and locomotor habit, based on 38 extant mammals. Mesotheriidae species (*C. munozi* † and *T. spegazzinianus* †) are shown separately for comparative purposes. LTL—lateral trochlear length (mm); MTL—medial trochlear length (mm); ATAA—astragalar tibial articular area (mm^2^); ANAA—astragalar navicular articular area (mm^2^); ANSL—astragalar navicular surface length (mm); ATTA—astragalar tibial tarsal angle (degrees); ACAA—astragalar calcaneal articular area (mm^2^). † indicates extinct species.

**Table 2:** Summary of astragalar functional indices across body mass, posture, top speed, and locomotor habit.

Biological Factors		TDI	NFP	TRAA	NRAA	CRAA
Body mass	Small (*n* = 6)	1.57 ± 0.31	1.49 ± 1.0	0.53 ± 0.05	0.16 ± 0.05	0.31 ± 0.10
	Medium (*n* = 20)	1.67 ± 0.43	3.22 ± 3.12	0.57 ± 0.08	0.14 ± 0.05	0.29 ± 0.07
	Large (*n* = 9)	2.02 ± 0.59	9.8 ± 4.57	0.47 ± 0.08	0.18 ± 0.10	0.35 ± 0.14
	Very large (*n* = 3)	2.01 ± 0.28	14.23 ± 8.55	0.42 ± 0.05	0.19 ± 0.12	0.39 ± 0.07
Posture	Plantigrade (*n* = 21)	1.59 ± 0.27	4.01 ± 4.27	0.52 ± 0.08	0.14 ± 0.06	0.34 ± 0.11
	Digitigrade (*n* = 10)	1.67 ± 0.49	2.86 ± 1.57	0.61 ± 0.05	0.12 ± 0.04	0.27 ± 0.03
	Unguligrade (*n* = 7)	2.44 ± 0.28	13.05 ± 5.74	0.44 ± 0.06	0.26 ± 0.05	0.3 ± 0.10
Top speed	Slow (*n* = 20)	1.70 ± 0.42	4.49 ± 5.66	0.52 ± 0.08	0.17 ± 0.08	0.31 ± 0.10
	Medium (*n* = 11)	1.90 ± 0.57	7.25 ± 5.36	0.49 ± 0.09	0.16 ± 0.07	0.35 ± 0.11
	Fast (*n* = 7)	1.75 ± 0.44	4.96 ± 4.97	0.61 ± 0.08	0.12 ± 0.05	0.27 ± 0.04
Locomotor habit	Arboreal (*n* = 13)	1.65 ± 0.25	1.94 ± 1.07	0.54 ± 0.06	0.16 ± 0.06	0.31 ± 0.09
	Cursorial (*n* = 8)	2.21 ± 0.47	12.86 ± 5.84	0.45 ± 0.07	0.23 ± 0.07	0.33 ± 0.11
	Scansorial (*n* = 4)	1.88 ± 0.43	5.38 ± 4.39	0.58 ± 0.10	0.15 ± 0.08	0.28 ± 0.03
	Terrestrial (*n* = 13)	1.57 ± 0.49	4.20 ± 3.74	0.56 ± 0.09	0.11 ± 0.05	0.33 ± 0.11
Mesotheridae †	*C. munozi* † (*n* = 1)	2.571	1.612	0.519	0.029	0.453
	*T. spegazzinianus* † (*n* = 1)	1.944	1.503	0.529	0.035	0.436

Mean ± standard deviation (*n* = number of species) of functional indices of the astragalus, grouped by body mass, limb posture, top speed, and locomotor habit, based on 38 extant mammals. Mesotheriidae species (*C. munozi* † and *T. spegazzinianus* †) are presented separately to explore potential differences in morphofunctional space. TDI—Trochlear Depth Index; NFP—Navicular Facet Proportions; TRAA—Trochlear Relative Articular Area; NRAA—Navicular Relative Articular Area; CRAA—Calcaneal Relative Articular Area. All indices are dimensionless and derived from astragalar morphometrics. † indicates extinct species.

**Table 3:** Summary of Total Angular Excursion (TAE, °), Summed Joint Angular Excursion (∑JAE, °), and Angular Efficiency Index (AEI, %) for forelimbs and hindlimbs of terrestrial mammals during the stance phase of walking, grouped by biological factors.

Biological Factors		Forelimb			Hindlimb		
		TAE (°)	∑ JAE (°)	AEI (%)	TAE (°)	∑ JAE (°)	AEI (%)
Body mass	Small	77 ± 17 (8)	124 ± 44 (15)	65.3 ± 21.9 (8)	78 ± 9 (15)	139 ± 56 (17)	56.4 ± 14.1 (15)
	Medium	60 ± 17 (21)	113 ± 58 (38)	53.8 ± 19.3 (21)	54 ± 13 (23)	95 ± 32 (57)	50.2 ± 17.0 (24)
	Large	52 ± 13 (20)	92 ± 45 (29)	54.7 ± 17.9 (20)	49 ± 15 (20)	84 ± 42 (39)	58.4 ± 14.7 (20)
	Very large	48 ± 10 (44)	79 ± 28 (53)	61.3 ± 14.3 (44)	42 ± 9 (44)	72 ± 25 (69)	57.4 ± 15.8 (44)
Posture	Plantigrade	64 ± 19 (22)	115 ± 45 (37)	56.4 ± 19.7 (22)	67 ± 15 (31)	119 ± 45 (47)	51.9 ± 16.2 (32)
	Digitigrade	57 ± 12 (28)	95 ± 52 (44)	53.3 ± 12.2 (28)	50 ± 12 (31)	82 ± 39 (47)	57.0 ± 14.2 (32)
	Unguligrade	47 ± 12 (30)	77 ± 27 (30)	65.2 ± 15.3 (30)	40 ± 8 (30)	69 ± 21 (58)	56.1 ± 16.2 (30)
Top speed	Slow	61 ± 20 (25)	111 ± 49 (44)	56.9 ± 20.0 (25)	62 ± 19 (33)	100 ± 45 (66)	54.8 ± 14.9 (33)
	Medium	57 ± 12 (27)	98 ± 41 (36)	55.2 ± 14.9 (27)	51 ± 12 (28)	97 ± 38 (43)	54.8 ± 13.1 (28)
	Fast	47 ± 11 (40)	79 ± 38 (52)	61.4 ± 16.6 (40)	43 ± 10 (40)	71 ± 28 (70)	58.5 ± 15.9 (40)
Locomotor habit	Arboreal	78 ± 21 (6)	106 ± 48 (18)	67.3 ± 15.0 (6)	79 ± 6 (12)	134 ± 50 (25)	49.0 ± 11.3 (12)
	Cursorial	48 ± 10 (39)	80 ± 38 (50)	62.8 ± 16.3 (39)	43 ± 11 (39)	71 ± 25 (68)	56.4 ± 13.7 (39)
	Scansorial	61 ± 16 (17)	113 ± 65 (23)	54.6 ± 21.1 (17)	55 ± 14 (19)	88 ± 39 (38)	61.7 ± 15.4 (19)
	Terrestrial	53 ± 13 (31)	102 ± 38 (44)	53.6 ± 14.9 (31)	50 ± 15 (32)	88 ± 33 (51)	54.0 ± 18.7 (33)

Mean values (± SD; n in parentheses) of Total Angular Excursion (TAE, °), Summed Joint Angular Excursion (∑JAE, °), and Angular Efficiency Index (AEI; TAE/∑JAE × 100, %) for forelimbs and hindlimbs of terrestrial mammals, grouped by biological factors: body mass, posture, top speed, and locomotor habit. These values were used as the comparative framework to estimate functional ranges for *Caraguatypotherium munozi* and *Trachytherus spegazzinianus*. For detailed descriptions of these indicators and the complete dataset, see the publicly available archive in Zenodo [24].

## Data Availability

The data supporting the findings of this study are available in both the main text and two supplementary files. Additional datasets are archived in publicly accessible Zenodo repositories: (1) Medina-González, P. (2025). *Supplementary Data for “Morphofunctional Spaces from the Astragalus: Exploring Angular Excursions and Mechanical Efficiency in Caraguatypotherium munozi (Notoungulata, Mesotheriidae)”* (Version 2.0.0) [dataset]. Zenodo. https://doi.org/10.5281/zenodo.16898870 [52]. This repository includes osteological measurements, functional indices, estimated body mass, and principal component analyses (PCA) of astragali from 38 extant mammals and mesotheriid species. (2) Medina González, P. (2025). *Supplementary Data for the Study on Joint Angular Excursion and Efficiency in Terrestrial Mammals* (Version 1.0.0) [dataset]. Zenodo. https://doi.org/10.5281/zenodo.15425733 [24]. This repository contains joint angular excursion (JAE), total angular excursion (TAE), and angular efficiency index (AEI) data for 182 extant terrestrial mammals, used as a comparative framework for estimating stance-phase ranges in mesotheriids.

## Supplementary Materials

The following supporting information can be downloaded at: https://www.mdpi.com/article/10.3390/biology14091290/s1, Supplementary File S1: Osteological measurements, functional indices, and estimated body mass of astragali from 38 extant mammals and mesotheriid species (*Caraguatypotherium munozi* and *Trachytherus spegazzinianus*), including definitions of all variables and biological factors. Supplementary File S2: Principal component analyses (PCA) of astragalar variables, including species morphospace positions, eigenvalues, and variable loadings, used to explore morphofunctional patterns and stance-phase locomotor strategies. References [17,49,50,51] are cited in the Supplementary Materials.

## Abbreviations

The following abbreviations are used in this manuscript:

ACAA	Astragalar Calcaneal Articular Area
AEI	Angular Efficiency Index
AMWT	Astragalar Maximum Width within the Trochlea
ANAA	Astragalar Navicular Articular Area
ANSL	Astragalar Navicular Surface Length
ATTA	Astragalar Tibial Tarsal Angle (also referred to as Head Orientation Angle, HOA)
CRAA	Calcaneal Relative Articular Area
ICC	Intraclass Correlation Coefficient
JAE	Joint Angular Excursion
LTL	Lateral Trochlear Length
MTL	Medial Trochlear Length
NFP	Navicular Facet Proportions
NRAA	Navicular Relative Articular Area
PCA	Principal Component Analysis
SD	Standard Deviation
SEM	Standard Error of Measurement
TAE	Total Angular Excursion
TDI	Trochlear Depth Index
TRAA	Trochlear Relative Articular Area
